# Prevalence of *Trypanosoma cruzi* Infection in Solid Organ Transplant Recipients: A Neglected Disease in America

**DOI:** 10.1093/ofid/ofae650

**Published:** 2024-11-08

**Authors:** German A Contreras, George Golovko

**Affiliations:** Division of Infectious Diseases, Department of Internal Medicine, University of Texas Medical Branch at Galveston, Galveston, Texas, USA; Department of Pharmacology & Toxicology, University of Texas Medical Branch at Galveston, Galveston, Texas, USA

**Keywords:** Chagas disease, reactivation, screening, solid organ transplant, *Trypanosoma cruzi*

## Abstract

This study investigates the prevalence of *Trypanosoma cruzi* infection among solid organ transplant recipients in the United States from 2019 to 2023 before transplantation. Utilizing data from a large multicenter network, we identified a rising seroprevalence of 4.8% from 1523 solid organ transplant recipients at the time of the evaluation for transplantation, particularly among lung and heart transplant recipients. The findings highlight the need for improved screening protocols to address this neglected tropical disease in transplant populations.

## Graphical Abstract

**Graphical abstract:**
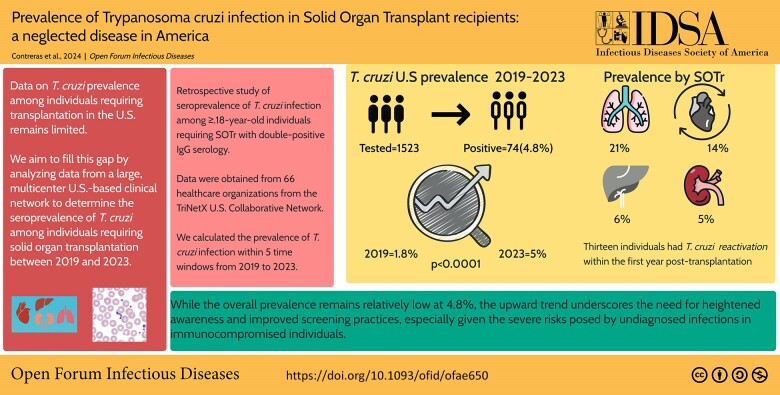
This graphical abstract is also available at Tidbit: https://tidbitapp.io/tidbits/prevalence-of-trypanosoma-cruzi-infection-in-solid-organ-transplant-recipients-a-neglected-disease-in-america-5effdb15-5cd2-4c40-8a17-8be39f09ff7d

## Teaser

This study reports a 4.8% prevalence of T. cruzi infection among 1,523 individuals who required solid organ transplantation from 2019 to 2023, with lung and heart transplant recipients showing the highest rates at the time of pre-transplant evaluation. These findings underscore the need for enhanced pre-transplant screening protocols.

Chagas disease, caused by the protozoan *Trypanosoma cruzi*, is a parasitic infection traditionally associated with rural areas of Latin America. However, due to increased migration from endemic regions, there is growing recognition of its presence in the United States [1, 2]. This migration has introduced new public health challenges, particularly for individuals undergoing solid organ transplantation, where undiagnosed *T. cruzi* infection can pose significant risks, including the potential for disease reactivation in an immunocompromised host [3–6].

Despite the increasing prevalence of Chagas disease in the United States, there remains a need for standardized pretransplant screening practices, especially in areas considered to have low endemicity. Previous studies have highlighted the importance of early detection and treatment to mitigate the risks of *T. cruzi* infection. Yet data on its prevalence among those requiring solid organ transplantation in the United States remain limited [7].

In this study, we aim to fill this gap by analyzing data from a large, multicenter, US-based clinical network to determine the seroprevalence of *T. cruzi* among individuals requiring solid organ transplantation between 2019 and 2023. We aim to underscore the need for more comprehensive screening protocols in individuals requiring solid organ transplantation by identifying the prevalence and associated demographic factors.

## METHODS

We conducted a retrospective study to evaluate the prevalence of *T. cruzi* infection among solid organ transplant recipients (SOTrs) in the United States during pretransplant evaluation. Data were obtained from the TriNetX US Collaborative Network. This clinical database aggregates de-identified electronic medical records (EMRs) from >86 million patients across 66 health care organizations (HCOs); the study period spanned from 2019 to 2023.

### Study Population

We included individuals aged 18 years or older who underwent heart, lung, kidney, or liver transplantation and had undergone double-positive immunoglobulin G (IgG) serology testing for *T. cruzi* during their pretransplant evaluation. The serological tests were conducted per World Health Organization (WHO) guidelines, with results reported as either positive or with an optical density (OD) of ≥1.20 [8]. The sensitivity and specificity of serological tests are typically around 85%–95% and 90%, respectively.

### Data Collection

Patient data were collected from the TriNetX platform, which harmonizes data across participating HCOs using standardized health care information exchange codes such as codes from the International Classification of Diseases, Tenth Edition (ICD-10), Healthcare Common Procedure Coding System (HCPCS), Current Procedural Terminology (CPT), and Health Level Seven (HL7). The diagnosis, procedures, and medication codes used in this study are described in detail in Supplementary Tables 1–4. We defined *T. cruzi* reactivation as the presence of a diagnosis of Chagas disease within the first year after transplantation. The ICD-10 codes used to define Chagas disease reactivation are described in Supplementary Table 3. Information regarding microscopic, histological examination, or quantitative polymerase chain reaction for *T. cruzi* diagnosis is unavailable on the TriNext platform. Follow-up continued until each patient had completed 1 year since transplantation.

Due to the platform's data harmonization process, all data were de-identified, and no protected health information was accessible. This study was approved by the University of Texas Medical Branch Institutional Review Board (IRB #20–0085), with IRB exemption granted for using de-identified data [9].

### Prevalence Calculation

We calculated the prevalence of *T. cruzi* infection among SOTrs within 5 discrete time windows from 2019 to 2023. For each time window, the prevalence was determined by identifying individuals with a positive serology result during the specified period. The lookback period was set to 1 day before the start of each time window to ensure comprehensive data capture.

### Statistical Analysis

Descriptive statistics were used to summarize the demographic and clinical characteristics of the study population. Continuous variables were presented as medians with interquartile ranges (IQRs), while categorical variables were presented as frequencies and percentages. Comparisons between groups were performed using chi-square (χ^2^) or Fisher exact tests, as appropriate. Prevalence analyses were conducted using the TriNetX platform, which provides built-in statistical tools for data. We carried out the chi-square test to assess if the change in prevalence by year, ethnicity, and sex over time was significant in Python. A *P* value <.05 was considered significant.

## RESULTS

We identified 1523 SOTrs tested for *T. cruzi* IgG serology during their pretransplant evaluation between 2019 and 2023. The cohort included recipients of kidney (n = 1102), liver (n = 165), heart (n = 69), lung (n = 49), and multiple organ transplants (n = 138). The population's median age (IQR) was 52 (26–74) years. Many patients were male (60%) and identified as non-Hispanic (49%) (Table 1). Among the 74 SOTrs who tested positive for *T. cruzi* before transplantation, 47% had a history of chronic ischemic heart disease, 26% had heart failure, 19% had a prior diagnosis of Chagas disease, and 18% had Chagas disease with heart involvement (Table 1).

**Table 1.** Baseline Demographic and Clinical Characteristics of Patients With Double-Positive *T. cruzi* Serology Before Transplantation

Demographics^a^	No. (%)
Total tested positive	74 (4.8)
Age, median (IQR), y	52.6 (26–74)
Male	50 (68.0)
Ethnicity	…
Hispanic	36 (48.6)
Not Hispanic	36 (48.6)
Race	…
White	31 (42.0)
Asian	14 (18.9)
Native American^b^	10 (13.5)
Black^b^	10 (13.5)
Pacific Islander^b^	10 (13.5)
Comorbidities	…
Chronic kidney disease	58 (78)
Hypertension	56 (76)
Type 2 diabetes mellitus	40 (54)
Chronic ischemic heart disease	35 (47)
Chronic liver disease	27 (36)
Obesity	27 (36)
Heart failure	19 (26)
Neoplasms	17 (23)
Chagas disease	14 (19)
Chagas disease with heart involvement	13 (18)

Abbreviation: IQR, interquartile range.

^a^Unless otherwise indicated, data are presented as the number/total number (%) of therapeutics.

^b^If the patient count is ≤10, results show the count as 10.

**Figure 1.**
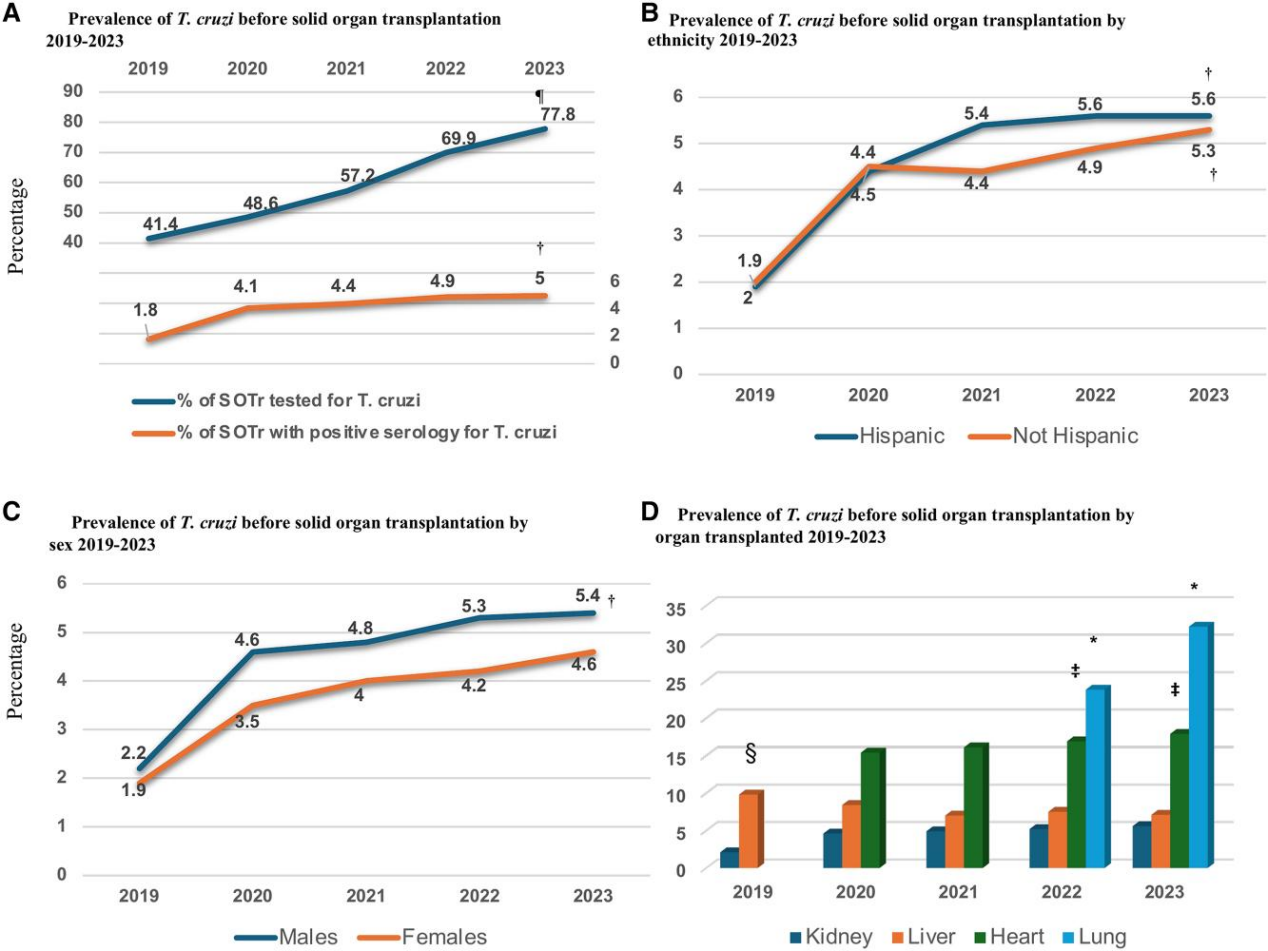
Prevalence of *T. cruzi* serology in a cohort of solid organ transplant recipients from 2019 to 2023. (*A–D*) describe the prevalence of *T. cruzi* by year of screening, ethnicity, sex and transplanted organ. ^a^2023 lung vs heart *P* = .004; lung vs liver *P* = .005; lung vs kidney *P* = .002; 2022 lung vs heart *P* = .004; lung vs liver *P* = .005; lung vs kidney *P* = .001. ^b^2023 heart vs kidney *P* = .021; 2022 heart vs kidney *P* = .021; 2021 heart vs kidney *P* = .021 and 2019 heart vs kidney *P* = .003. ^c^2019 liver vs kidney *P* = .003. ^d^*P* < .0001. ^e^*P* = .043.

The overall prevalence of positive *T. cruzi* serology in this cohort was 4.8%, with a significant increase in prevalence from 2019 to 2023 (Figure 1*A*) (*P* < .0001). This progressive increase in prevalence was also driven by a significant progressive rise in infection screening (*P* = .043) (Figure 1*A*). Geographically, most positive cases were detected in the Western United States, with Hispanic individuals and males showing a significantly higher likelihood of having a positive *T. cruzi* serology throughout the observation period (Figure 1*B* and *C*). A breakdown by organ type revealed that lung (21%) and heart (14%) transplant recipients had the highest prevalence of positive serology compared with liver (6%) and kidney (5%) recipients (Figure 1*D*). Thirteen patients (17.6%) were diagnosed with *T. cruzi* reactivation within the first year after transplantation, with a median (IQR) of 8 (2–4) months. All reactivation cases were treated with benznidazole, and there were no deaths attributed to Chagas disease following treatment.

## DISCUSSION

Our study highlights the increasing prevalence of *T. cruzi* infection among solid organ transplant recipients in the United States from 2019 to 2023, with a notable rise observed, particularly among lung and heart transplant recipients. While the overall prevalence remains relatively low at 4.8%, the upward trend underscores the need for heightened awareness and improved screening practices, especially given the severe risks posed by undiagnosed infections in immunocompromised individuals [10–14].

The demographic trends observed in our study are consistent with prior research, showing a higher prevalence of positive *T. cruzi* serology among Hispanic individuals and males [10, 11]. This demographic association likely reflects the migration patterns from endemic regions, emphasizing the importance of considering patient origin and ethnicity in the risk assessment for Chagas disease. The higher prevalence rates in lung and heart transplant recipients could be attributed to existing comorbidities, such as chronic ischemic heart disease and heart failure, which are known to be associated with Chagas disease.

Our findings on *T. cruzi* reactivation rates post-transplantation provide essential insights into the clinical management of these patients. Although the reactivation rate was relatively low at 17.6%, it is critical to monitor these patients closely, as reactivation can lead to severe complications [3]. The successful treatment of all reactivation cases with benznidazole in our cohort suggests that early detection and timely intervention can effectively mitigate the risks associated with reactivation. However, the absence of deaths related to Chagas disease in this study does not diminish the potential severity of the disease if left untreated.

Despite these important findings, our study has several limitations that warrant cautious interpretation. The retrospective design and reliance on de-identified data from the TriNetX platform limit our ability to access detailed patient histories (ie, benznidazole prophylaxis) and specific serological test information. Trinext rounds to 10 if a patient or group has a number of events ≤10. The platform does this to safeguard protected health information, and because of this, we were able to determine the distribution of *T cruzi* reactivation by organ transplanted. The potential for selection bias exists due to the variable data update schedules across participating health care organizations. During this process, TriNetX carries out an extensive data quality assessment and harmonization. The platform rejects records that do not meet the TriNetX quality standards, thereby dropping data from transplant centers. Chagas disease reactivation might have been overestimated in our study by using ICD-10 codes instead of molecular or histopathological data that support the diagnosis of *T. cruzi* infection. Yet, our reactivation prevalence is similar to what is reported in the literature [3]. Given these limitations, future research should aim to include more granular data on patient ethnicity, migration history, and geographic origin to better understand the risk factors for *T. cruzi* infection and the rising prevalence among non-Hispanics observed in our cohort. Moreover, multicenter prospective studies with standardized screening protocols could provide a more comprehensive understanding of the disease burden and inform the development of targeted interventions.

In conclusion, our study underscores the need for expanded screening and awareness of Chagas disease among transplant candidates in the United States. As the prevalence of *T. cruzi* infection continues to rise, particularly in specific demographic groups, health care providers must remain vigilant in identifying and managing this neglected tropical disease. Prospective interventional studies are needed to assess the cost-effectiveness of screening all patients requiring transplantation, regardless of their ethnicity or geographic origin, to mitigate the risks associated with undiagnosed infection, given the growing prevalence in non-Hispanic individuals in our study. In the meantime, enhanced education and training for clinicians, coupled with the implementation of standardized screening protocols, will be crucial in ensuring the health and safety of transplant recipients.

## Supplementary Material

ofae650_Supplementary_Data

## Supplementary Data

Supplementary materials are available at *Open Forum Infectious Diseases* online. Consisting of data provided by the authors to benefit the reader, the posted materials are not copyedited and are the sole responsibility of the authors, so questions or comments should be addressed to the corresponding author.
