# Supplementary material for: Prevalence of Trypanosoma cruzi Infection in Solid Organ Transplant Recipients: A Neglected Disease in America
Source: Open Forum Infect Dis. 2024 Nov 8;11(12):ofae650. doi: 10.1093/ofid/ofae650 (PMC11600953; doi:10.1093/ofid/ofae650)
Supplement: ofae650_Supplementary_Data [file ofae650_supplementary_data.docx]

Supplemental Table 1 ICD10 and CPT codes used to build study cohort

| **UMLS:ICD10CM:Z94.0** | **Kidney transplant status** |
| --- | --- |
| **UMLS:CPT:00868** | Anesthesia for extraperitoneal procedures in lower abdomen, including urinary tract; renal transplant (recipient) |
| **UMLS:CPT:1008098** | Renal Transplantation Procedures |
| **UMLS:CPT:1008109** | Renal allotransplantation, implantation of graft |
| **UMLS:CPT:50360** | Renal allotransplantation, implantation of graft; without recipient nephrectomy |
| **UMLS:CPT:50365** | Renal allotransplantation, implantation of graft; with recipient nephrectomy |
| **UMLS:ICD10PCS:0TY0** | Urinary System / Transplantation / Kidney, Right |
| **UMLS:ICD10PCS:0TY00Z0** | Transplantation of Right Kidney, Allogeneic, Open Approach |
| **UMLS:ICD10PCS:0TY00Z1** | Transplantation of Right Kidney, Syngeneic, Open Approach |
| **UMLS:ICD10PCS:0TY00Z2** | Transplantation of Right Kidney, Zooplastic, Open Approach |
| **UMLS:ICD10PCS:0TY1** | Urinary System / Transplantation / Kidney, Left |
| **UMLS:ICD10PCS:0TY10Z0** | Transplantation of Left Kidney, Allogeneic, Open Approach |
| **UMLS:ICD10PCS:0TY10Z1** | Transplantation of Left Kidney, Syngeneic, Open Approach |
| **UMLS:ICD10PCS:0TY10Z2** | Transplantation of Left Kidney, Zooplastic, Open Approach |
| **UMLS:CPT:00796** | Anesthesia for intraperitoneal procedures in upper abdomen including laparoscopy; liver transplant (recipient) |
| **UMLS:CPT:1007811** | Liver Transplantation Procedures |
| **UMLS:CPT:47135** | Liver allotransplantation, orthotopic, partial or whole, from cadaver or living donor, any age |
| **UMLS:ICD10CM:Z94.4** | Liver transplant status |
| **UMLS:ICD10PCS:0FY0** | Hepatobiliary System And Pancreas / Transplantation / Liver |
| **UMLS:ICD10PCS:0FY00Z0** | Transplantation of Liver, Allogeneic, Open Approach |
| **UMLS:ICD10PCS:0FY00Z1** | Transplantation of Liver, Syngeneic, Open Approach |
| **UMLS:ICD10PCS:0FY00Z2** | Transplantation of Liver, Zooplastic, Open Approach |
| **UMLS:CPT:32854** | Lung transplant, double (bilateral sequential or en bloc); with cardiopulmonary bypass |
| **UMLS:CPT:32853** | Lung transplant, double (bilateral sequential or en bloc); without cardiopulmonary bypass |
| **UMLS:CPT:32851** | Lung transplant, single; without cardiopulmonary bypass |
| **UMLS:CPT:32852** | Lung transplant, single; with cardiopulmonary bypass |
| **UMLS:CPT:1006036** | Lung Transplantation Procedures |
| **UMLS:CPT:1006038** | Lung transplant, single |
| **UMLS:CPT:1006041** | Lung transplant, double (bilateral sequential or en bloc) |
| **UMLS:CPT:1006332** | Heart/Lung Transplantation Procedures |
| **UMLS:CPT:00580** | Anesthesia for heart transplant or heart/lung transplant |
| **UMLS:ICD10CM:Z94.2** | Lung transplant status |
| **UMLS:ICD10PCS:0BYC** | Respiratory System / Transplantation / Upper Lung Lobe, Right |
| **UMLS:ICD10PCS:0BYC0Z0** | Transplantation of Right Upper Lung Lobe, Allogeneic, Open Approach |
| **UMLS:ICD10PCS:0BYC0Z1** | Transplantation of Right Upper Lung Lobe, Syngeneic, Open Approach |
| **UMLS:ICD10PCS:0BYC0Z2** | Transplantation of Right Upper Lung Lobe, Zooplastic, Open Approach |
| **UMLS:ICD10PCS:0BYD** | Respiratory System / Transplantation / Middle Lung Lobe, Right |
| **UMLS:ICD10PCS:0BYD0Z0** | Transplantation of Right Middle Lung Lobe, Allogeneic, Open Approach |
| **UMLS:ICD10PCS:0BYD0Z1** | Transplantation of Right Middle Lung Lobe, Syngeneic, Open Approach |
| **UMLS:ICD10PCS:0BYD0Z2** | Transplantation of Right Middle Lung Lobe, Zooplastic, Open Approach |
| **UMLS:ICD10PCS:0BYF** | Respiratory System / Transplantation / Lower Lung Lobe, Right |
| **UMLS:ICD10PCS:0BYF0Z0** | Transplantation of Right Lower Lung Lobe, Allogeneic, Open Approach |
| **UMLS:ICD10PCS:0BYF0Z1** | Transplantation of Right Lower Lung Lobe, Syngeneic, Open Approach |
| **UMLS:ICD10PCS:0BYF0Z2** | Transplantation of Right Lower Lung Lobe, Zooplastic, Open Approach |
| **UMLS:ICD10PCS:0BYG** | Respiratory System / Transplantation / Upper Lung Lobe, Left |
| **UMLS:ICD10PCS:0BYG0Z0** | Transplantation of Left Upper Lung Lobe, Allogeneic, Open Approach |
| **UMLS:ICD10PCS:0BYG0Z1** | Transplantation of Left Upper Lung Lobe, Syngeneic, Open Approach |
| **UMLS:ICD10PCS:0BYG0Z2** | Transplantation of Left Upper Lung Lobe, Zooplastic, Open Approach |
| **UMLS:ICD10PCS:0BYH** | Respiratory System / Transplantation / Lung Lingula |
| **UMLS:ICD10PCS:0BYH0Z0** | Transplantation of Lung Lingula, Allogeneic, Open Approach |
| **UMLS:ICD10PCS:0BYH0Z1** | Transplantation of Lung Lingula, Syngeneic, Open Approach |
| **UMLS:ICD10PCS:0BYH0Z2** | Transplantation of Lung Lingula, Zooplastic, Open Approach |
| **UMLS:ICD10PCS:0BYJ** | Respiratory System / Transplantation / Lower Lung Lobe, Left |
| **UMLS:ICD10PCS:0BYJ0Z0** | Transplantation of Left Lower Lung Lobe, Allogeneic, Open Approach |
| **UMLS:ICD10PCS:0BYJ0Z1** | Transplantation of Left Lower Lung Lobe, Syngeneic, Open Approach |
| **UMLS:ICD10PCS:0BYJ0Z2** | Transplantation of Left Lower Lung Lobe, Zooplastic, Open Approach |
| **UMLS:ICD10PCS:0BYK** | Respiratory System / Transplantation / Lung, Right |
| **UMLS:ICD10PCS:0BYK0Z0** | Transplantation of Right Lung, Allogeneic, Open Approach |
| **UMLS:ICD10PCS:0BYK0Z1** | Transplantation of Right Lung, Syngeneic, Open Approach |
| **UMLS:ICD10PCS:0BYK0Z2** | Transplantation of Right Lung, Zooplastic, Open Approach |
| **UMLS:ICD10PCS:0BYL** | Respiratory System / Transplantation / Lung, Left |
| **UMLS:ICD10PCS:0BYL0Z0** | Transplantation of Left Lung, Allogeneic, Open Approach |
| **UMLS:ICD10PCS:0BYL0Z1** | Transplantation of Left Lung, Syngeneic, Open Approach |
| **UMLS:ICD10PCS:0BYL0Z2** | Transplantation of Left Lung, Zooplastic, Open Approach |
| **UMLS:ICD10PCS:0BYM** | Respiratory System / Transplantation / Lungs, Bilateral |
| **UMLS:ICD10PCS:0BYM0Z0** | Transplantation of Bilateral Lungs, Allogeneic, Open Approach |
| **UMLS:ICD10PCS:0BYM0Z1** | Transplantation of Bilateral Lungs, Syngeneic, Open Approach |
| **UMLS:ICD10PCS:0BYM0Z2** | Transplantation of Bilateral Lungs, Zooplastic, Open Approach |
| **UMLS:CPT:33929** | Removal of a total replacement heart system (artificial heart) for heart transplantation (List separately in addition to code for primary procedure) |
| **UMLS:CPT:33935** | Heart-lung transplant with recipient cardiectomy-pneumonectomy |
| **UMLS:CPT:33945** | Heart transplant, with or without recipient cardiectomy |
| **UMLS:CPT:00580** | Anesthesia for heart transplant or heart/lung transplant |
| **UMLS:CPT:1006332** | Heart/Lung Transplantation Procedures |
| **UMLS:CPT:33929** | Removal of a total replacement heart system (artificial heart) for heart transplantation (List separately in addition to code for primary procedure) |
| **UMLS:CPT:33935** | Heart-lung transplant with recipient cardiectomy-pneumonectomy |
| **UMLS:CPT:33945** | Heart transplant, with or without recipient cardiectomy |
| **UMLS:ICD10CM:Z94.1** | Heart transplant status |
| **UMLS:ICD10CM:Z94.3** | Heart and lungs transplant status |
| **UMLS:ICD10PCS:02YA** | Heart And Great Vessels / Transplantation / Heart |
| **UMLS:ICD10PCS:02YA0Z0** | Transplantation of Heart, Allogeneic, Open Approach |
| **UMLS:ICD10PCS:02YA0Z1** | Transplantation of Heart, Syngeneic, Open Approach |
| **UMLS:ICD10PCS:02YA0Z2** | Transplantation of Heart, Zooplastic, Open Approach |

Supplemental Table 2 LOINC codes used chagas infection serological testing

| **UMLS:LNC:23785-9** | **Trypanosoma cruzi Ab [Presence] in Serum** |
| --- | --- |
| **UMLS:LNC:32725-4** | Trypanosoma cruzi IgG Ab [Presence] in Serum |
| **UMLS:LNC:14094-7** | Trypanosoma cruzi IgG Ab [Titer] in Serum |
| **UMLS:LNC:60553-5** | Trypanosoma cruzi Ab [Presence] in Serum from Donor |
| **UMLS:LNC:13291-0** | Trypanosoma cruzi IgG Ab [Units/volume] in Serum |
| **UMLS:LNC:13290-2** | Trypanosoma cruzi IgM Ab [Units/volume] in Serum |
| **UMLS:LNC:57320-4** | Trypanosoma cruzi Ab [Presence] in Serum or Plasma by Immunoassay |
| **UMLS:LNC:59387-1** | Trypanosoma cruzi IgG Ab [Units/volume] in Serum by Immunoassay |

Supplemental Table 3 ICD-10 codes used to assess reactivation after organ transplantation was performed

| **UMLS:ICD10CM:B57.0** | **Acute Chagas' disease with heart involvement** |
| --- | --- |
| **UMLS:ICD10CM:B57.1** | Acute Chagas' disease without heart involvement |
| **UMLS:ICD10CM:B57.2** | Chagas' disease (chronic) with heart involvement |
| **UMLS:ICD10CM:B57.3** | Chagas' disease (chronic) with digestive system involvement |
| **UMLS:ICD10CM:B57.5** | Chagas' disease (chronic) with other organ involvement |

Supplemental Table 4 Medications codes used to assess treatment for *T. cruzi* reactivation after organ transplantation was performed

| **NLM:RXNORM:18994** | **benznidazole** |
| --- | --- |
| **NLM:RXNORM:7421** | nifurtimox |
